# Fast-track development of vaccines for SARS-CoV-2: The shots that saved the world

**DOI:** 10.3389/fimmu.2022.961198

**Published:** 2022-10-03

**Authors:** Vivek P. Chavda, Qian Yao, Lalitkumar K. Vora, Vasso Apostolopoulos, Chirag A. Patel, Rajashri Bezbaruah, Aayushi B. Patel, Zhe-Sheng Chen

**Affiliations:** ^1^ Department of Pharmaceutics and Pharmaceutical Technology, LM College of Pharmacy, Ahmedabad, Gujarat, India; ^2^ Graduate School, University of St. La Salle, Bacolod City, Philippines; ^3^ School of Pharmacy, Queen’s University Belfast, Belfast, United Kingdom; ^4^ Institute for Health and Sport, Victoria University, Melbourne, VIC, Australia; ^5^ Department of Pharmacology, LM College of Pharmacy, Ahmedabad, Gujarat, India; ^6^ Department of Pharmaceutical Sciences, Faculty of Science and Engineering, Dibrugarh University, Dibrugarh, Assam, India; ^7^ Pharmacy Section, LM. College of Pharmacy, Ahmedabad, Gujarat, India; ^8^ Department of Pharmaceutical Science, College of Pharmacy and Health Sciences, St. John’s University, New York, NY, United States

**Keywords:** COVID-19 outbreak, SARS-CoV-2 variants, vaccine, variant of concern, omicron variant, delta variant

## Abstract

In December 2019, an outbreak emerged of severe acute respiratory syndrome coronavirus 2 (SARS-CoV-2) which leads to coronavirus disease 2019 (COVID-19). The World Health Organisation announced the outbreak a global health emergency on 30 January 2020 and by 11 March 2020 it was declared a pandemic. The spread and severity of the outbreak took a heavy toll and overburdening of the global health system, particularly since there were no available drugs against SARS-CoV-2. With an immediate worldwide effort, communication, and sharing of data, large amounts of funding, researchers and pharmaceutical companies immediately fast-tracked vaccine development in order to prevent severe disease, hospitalizations and death. A number of vaccines were quickly approved for emergency use, and worldwide vaccination rollouts were immediately put in place. However, due to several individuals being hesitant to vaccinations and many poorer countries not having access to vaccines, multiple SARS-CoV-2 variants quickly emerged that were distinct from the original variant. Uncertainties related to the effectiveness of the various vaccines against the new variants as well as vaccine specific-side effects have remained a concern. Despite these uncertainties, fast-track vaccine approval, manufacturing at large scale, and the effective distribution of COVID-19 vaccines remain the topmost priorities around the world. Unprecedented efforts made by vaccine developers/researchers as well as healthcare staff, played a major role in distributing vaccine shots that provided protection and/or reduced disease severity, and deaths, even with the delta and omicron variants. Fortunately, even for those who become infected, vaccination appears to protect against major disease, hospitalisation, and fatality from COVID-19. Herein, we analyse ongoing vaccination studies and vaccine platforms that have saved many deaths from the pandemic.

## Introduction

The COVID-19 pandemic had spread to 228 countries as of September 15, 2022 (plus outbreaks in 2 cruise ships), with over 615.5 million laboratory-confirmed cases and 6.52 million deaths causing considerable social and economic devastation. The main strategy used against COVID-19 is to alleviate symptoms, hospitalisations and deaths. As such, several therapeutic approaches are being evaluated for their effectiveness in reducing/eliminating SARS-CoV-2 viral load and reducing symptoms (1–4). Initially, the strategy was to repurpose existing therapeutics to allow for faster drug development (2, 5, 6). Several clinical trials on existing drugs have been completed, and others are in progress (4, 7–9). Despite the numerous approaches, there have been repeated cases where recovered patients are reinfected, highlighting the need for herd immunity against SARS-CoV-2 (10). However, drugs such as molnupiravir, bamlanivimab, bebtelovimab, and tixagevimab with cilgavimab work well and are predominantly used in elderly and high-risk patients (2, 3, 11). Patients with comorbidities such as cardiovascular disease, chronic respiratory disease, hypertension, type-2 diabetes, and cancer may be affected with unfavorable outcomes (12). Furthermore, the emergence of monkey pox, Langya virus and tomato flu a manifestation of hand, foot and mouth diseases complicates the COVID-19 management process (13–16). SARS-CoV-2 manifestations are more serious in the elderly and those with chronic diseases, owing to the overactive and persistent innate inflammatory processes and anatomical and functional alterations in the physiological systems (17). This will impede not only the capability to defend against respiratory infections but also the ability to build efficient vaccination defense. According to Elizabeth C Stahl (18), “The function of lymphoid and nonlymphoid tissues involved in the host immune response declines with age. The production of naive T and B-lymphocytes is decreased when primary lymphoid organs degenerate, resulting in diminished transition to secondary lymphoid organs and antigen encounter sites. In addition, proinflammatory cells and mediators may accumulate in the lungs and extrapulmonary organs. Protective immunity to vaccination is low or faulty in elderly individuals, while autoimmunity rises. As a result, while developing SARS-CoV-2 vaccines, it will be crucial to keep in mind that older individuals may not react to vaccinations as well as younger people (19).”

**Figure 1:**
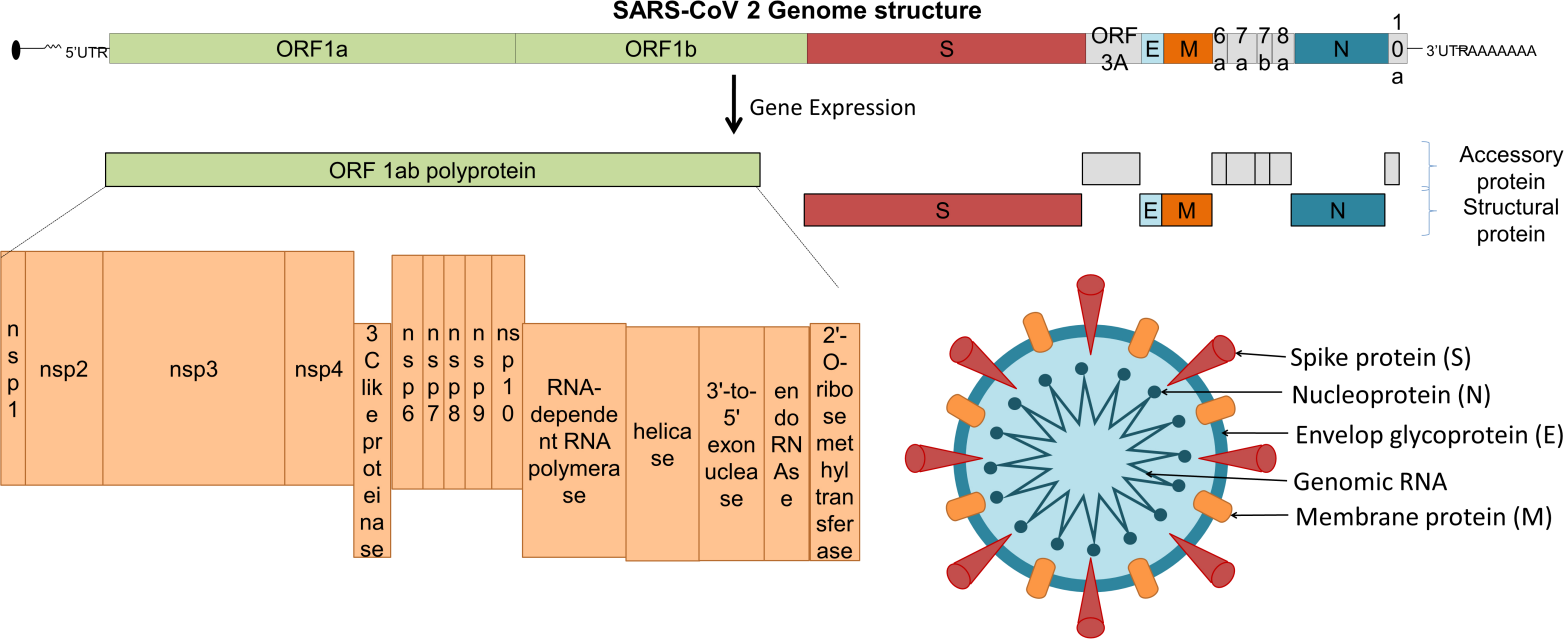
SARS-CoV-2 structural proteins and genomic organization.

The viral structural proteins and genomic organization is summarized in **Figure 1**. The immune system plays a critical role in the pathogenesis of COVID-19 as well as vaccine effectiveness. To design a safe and effective vaccine, preclinical and clinical trials are conducted with caution to minimise adverse reactions (20). Herein, we discuss different targets and platforms that are currently being used as COVID-19 vaccine candidates, the effects of variants on vaccine efficacy, as well as the issues connected with fast-tracking of vaccines and vaccination ethics (21).

### SARS-CoV-2 mutations and impact

**Figure 2:**
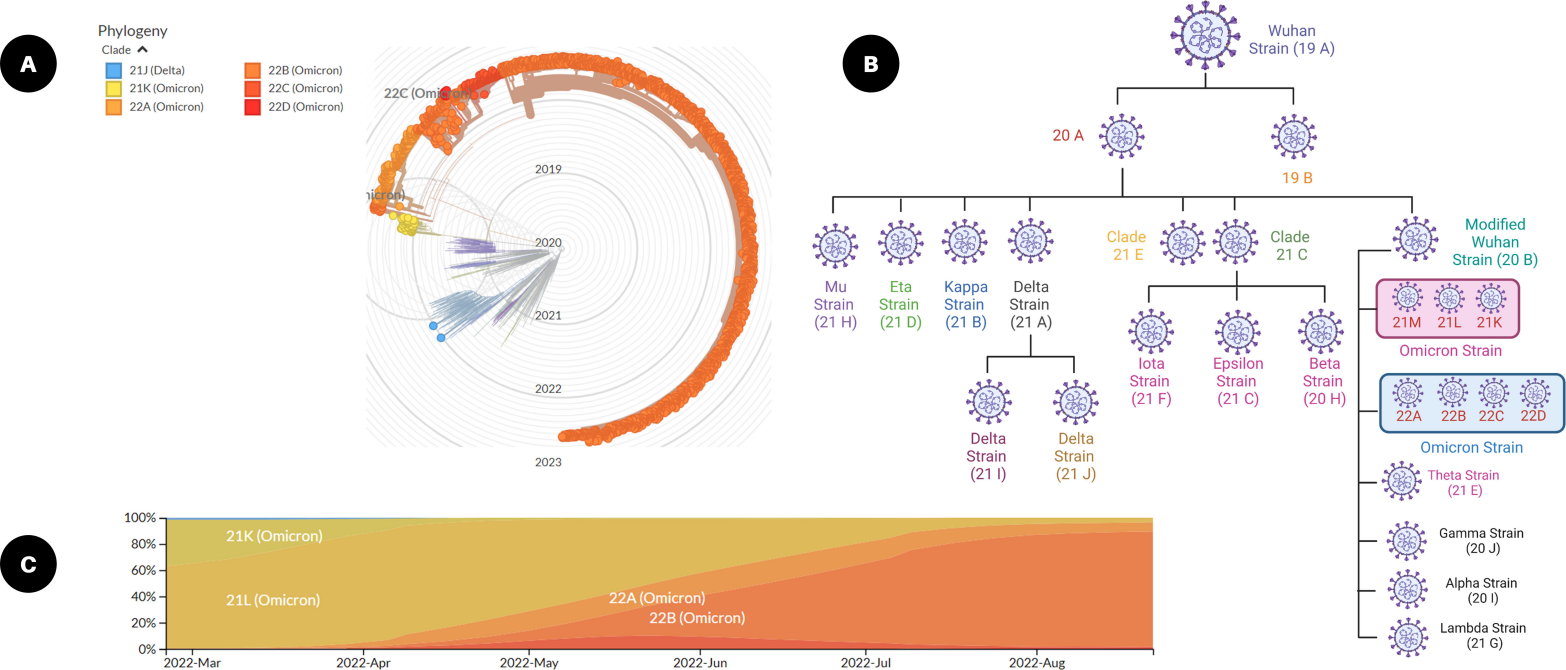
SARS-CoV-2 mutations and viral variants. **(A)** Radial graph of emerging SARS-CoV-2 variants from December 2021 to August 2022 (built with nextstrain/ncov). **(B)** Frequencies of clades from March 2022 to August 2022 (from nextstrain.org), and **(C)** classification of SARS-CoV-2 omicron variant based on a clade tree (created with Biorender.com).

Viral mutations arise when the genetic sequences of the genome that make up a virus change. This virus survival instinct of generating thousands of copies of themselves over time leads to errors in the genetic fingerprint as a part of the natural process. These random genetic drifts may lead to no change or may increase or decrease the virulent capability to spread or cause diseases (22, 23). While maximum viral mutations in the viral genome are mostly point mutations with a deleted arm or neutral mutations, a small percentage will alter functional viral parts and may modify contagiousness or infection severity. The viral genomic evolution or SARS-CoV-2 remained largely unchanged for approximately 11 months after its emergence in late 2019. Later, in 2020 and thereafter, genomic evolution was pigeonholed by the advent mutation series, and what emerged was what is commonly known as ‘variants of concern (VOC)’ (**Figure 2**). These mutations affect some virus attributes, as well as antigenicity and transmissibility. There is unfavorable evidence of the lowered neutralization capability of some coronavirus variants against the vaccine, which may lead to many uncertainties in effective vaccine development. A possible solution to this is by updating the vaccine sequences considering major mutations of the emerging variants and their interrelationships (24).

The World Health Organization (WHO) demonstrated three types of SARS-CoV-2 variants in collaboration with a SARS-CoV-2 interagency group as (i) variant of interest (VOI), (ii) variant of concern (VOC), and (iii) variant of high consequence (VOHC). VOIs include zeta (P.2), theta (P.3), epsilon (B.1.427, B.1.429), kappa (B.1.617.1), iota (B.1.526), eta (B.1.525), lambda (C.37) and the mu variant (B.1621) (25). The WHO changed the designation of the following VOCs to, ‘previously circulating VOCs’ - the alpha variant (B.1.1.7), beta (B.1.351), gamma variant (P.1), and delta variant (B.1.617.2) (26). The omicron is the current VOC and some sub-variants are classified as, ‘under monitoring’ (B.1.1.529, BA.1, BA.1.1, BA.2, BA.2.12.1, BA.2.75, BA.3, BA.4, and BA.5) (**Table 1**). No SARS-CoV-2 variants have been classified as VOHC (22, 39).

**Table 1:** SARS-CoV-2 variants, mutations and their impact.

WHO variant name	Sub variants	Date first identified/Country	Spike protein mutations	Other mutations	Impact	Ref.
Alpha	B.1.1.7	November 2020/UK	HV69-70del, Y144del, N501Y, A570D, D614G, P681H, T716I, S982A, D1118H	D3L, R203K, G204R, S235F	• Increased transmission • Increase in incidences of hospitalisations	(27)
Beta	B.1.351	May 2020/South Africa	L18F, D80A, D215G, 242-244del, R246I, K417N, E484K, N501Y, D614G, A701V	T205I	• Increased transmission	(28)
Gamma	B.1.1.248 (P.1 and P.2)	Nov-2020/Brazil	K417T, E484K, and N501Y	D138Y, R190S	• Increase affinity to ACE 2 receptor • Reduces the neutralizing activity	(29, 30)
Delta	B.1.617.2 (Delta)	October 2020/India	T19R, G142D, EF156-157del, R158G, L452R, T478K, D614G, P681R, D950N	D63G, R203M, D377Y	• Decreased ability of immune system to identify the virus. • Increase affinity to ACE 2 receptor	(31)
	(Delta plus)	03 March 2022/India	K417N, T19R, G142D, EF156-157del, R158G, L452R, T478K, D614G, P681R, D950N	D63G, R203M, D377Y	• It has higher tolerance to monoclonal antibodies compare to delta variant • Increase transmissibility and higher binding affinity towards lung epithelial cells compare to all other variants.	(32)
Omicron	BA.1	08 November 2021/ Botswana, South Africa	A67V, HV69-70del, T95I, G142D, VYY143-145del, N211del, L212I, ins214EPE, G339D, S371L, S373P, S375F, K417N, N440K, G446S, S477N, T478K, E484A, Q493R, G496S, Q498R, N501Y, Y505H, T547K, D614G, H655Y, N679K, P681H, N764K, D796Y, N856K, Q954H, N969K, L981F	nsp3 (K38R, V1069I, Δ1265, L1266I, A1892T), nsp4 (T492I), nsp5 (P132H), nsp6 (Δ105-107, A189 V), nsp12 (P323L), and nsp14 (I42V). Nsp3 (Plpro) and nsp5 (3Clpro, main protease)	• Changes in the shape of protein to which different class of antibody binds. • Compare to other variants has the highest transmission	(23, 33–35)
	BA.2	22 October 2021/Philippines	BA.2 shares 32 mutations with BA.1 but has 28 distinct ones. RBD mutations: 371F, T376A, D405N, and R408S, and BA.3 has S371F, D405N, and G446S		• All these variants have higher transmission and stronger immune invasion compare to BA.1	(36–38)
	BA.3	23 November 2021/northwest South Africa	N501Y, Q498R, H655Y, N679K, and P681H. BA.3 shares ten mutations (A67V, H69del, V70del, T95I, V143del, Y144del, Y145del, N211I, L212del, and G446S) from BA.1 and two (S371F and D405N) mutations from BA.2 to form its spike protein.			
	BA.4	12 May 2022/South Africa, Botswana, Denmark, UK	S:del69/70, S:L452R, S:F486V, S:Q493R reversion			
	BA.5	25 May 2022/Portugal	BA.2-like constellation in the spike protein + S:del69/70, S:L452R, S:F486V, S:Q493R reversion			

**Figure 3:**
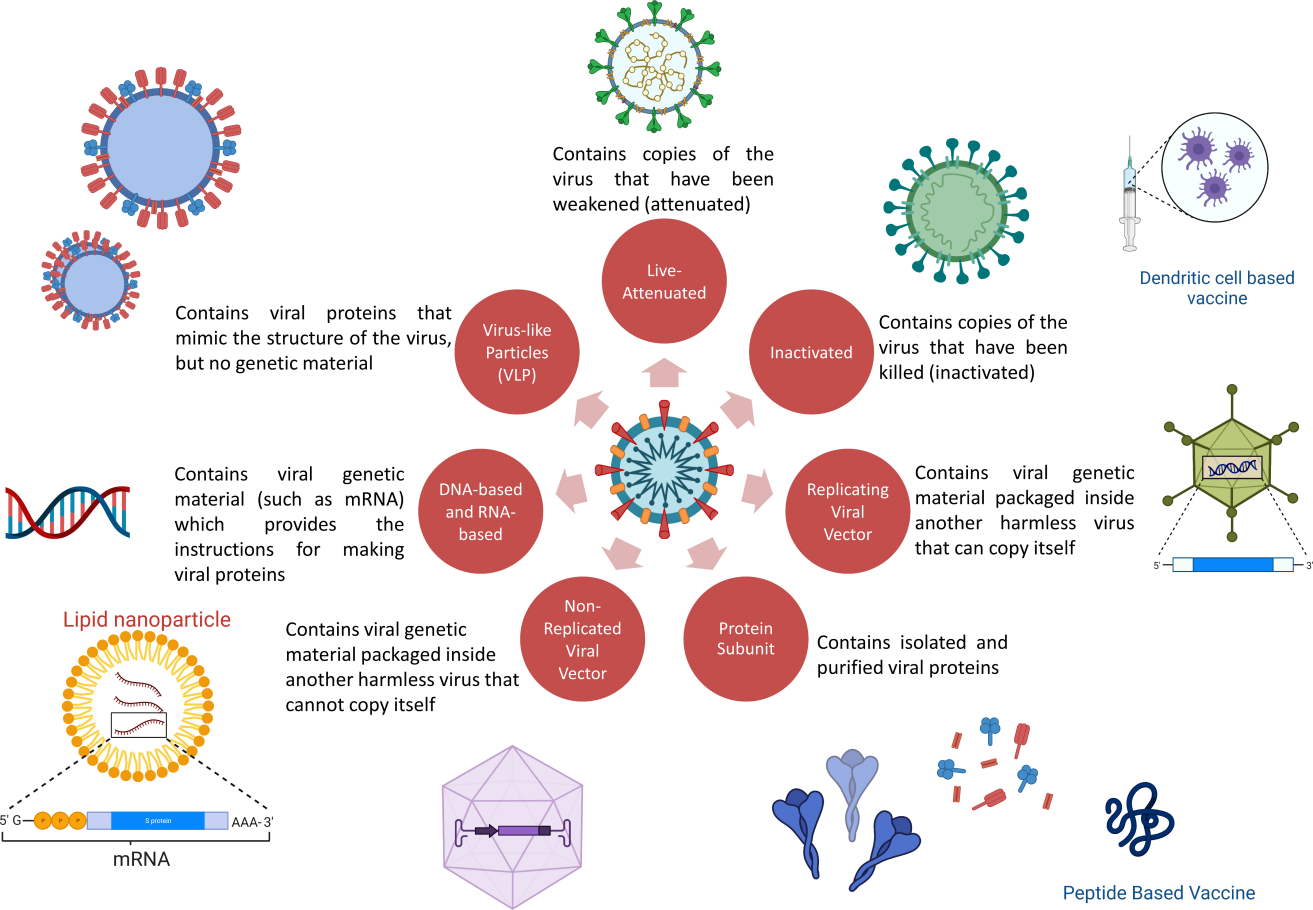
Hybrid variants of SARS-CoV-2 (Created with BioRender.com).

Most variations in the structure of viruses occurs due to mutation in the genetic material, but recently, due to recombination of two different variants or sub variants, the generation of recombinant variants have been detected. Recombinant variants, also known as hybrid variants, surfaced due to the transfer of genetic material between two viral variants. Recently, in some countries, such as France and Europe, very few cases of recombinant variants have been observed. The WHO designated these variants as XE and XF (**Figure 3**). Early 2022, a new recombinant variant referred to as “deltacron”, also known as XD was identified which is the combination of omicron and delta variants. An infection of both variants in a single patient was observed in France in January 2022 (40). The delta variant is the most dangerous variant among all variants of COVID-19. The delta variant is responsible for most deaths across the world, and omicron is the most infectious variant among all variants. Deltacron is known as BA.1 x AY.4, where BA.1 is an omicron variant and AY.4 is a delta variant (41). In the United Kingdom, a combination of two subvariants named BA.1 and BA.2 (also known as stealth omicron) of the omicron variant was noted in one patient (XE variant). In the case of the XE variant, most of its structure, including the spike protein, is of the BA.2 sub variant, but the 5′ part of its structure is made up of the BA.1 sub variant (42). In addition, XF, is the result of the combination of BA.1 subvariant of omicron and delta variants (43). Currently, five main sublineages of omicron variants have been detected (BA.1 to BA.5). The BA.4 and BA.5 lineages managed to dominate the planet between April-July 2022 since their emergence, whereas BA.2 began to dwindle at almost the same period (44).

Compared to other RNA viruses, the activation and variability of the SARS-CoV-2 3’-5’ exonuclease during viral genome transcription can lead to changes in non-structural protein (nsp)14 (45). Even a single point mutation may impact viral proteins and their physicochemical nature, which could consequently modify the binding affinity of the virus with host cells (46, 47). Importantly, the emergence of these amino acid mutations may result in an antigenic drift that reduces the neutralization ability of the immune serum from vaccines, causing a new wave of mutations of the SARS-CoV-2 spike protein. Despite the fact that the cumulative mutation rates of the SARS-CoV-2 genome are lower than those of influenza and HIV-1 viruses, the current advent of a spreading mutant strain has raised concerns regarding the efficacy of COVID-19 vaccines (48).

## Vaccine platforms against SARS-CoV-2

**Figure 4:**
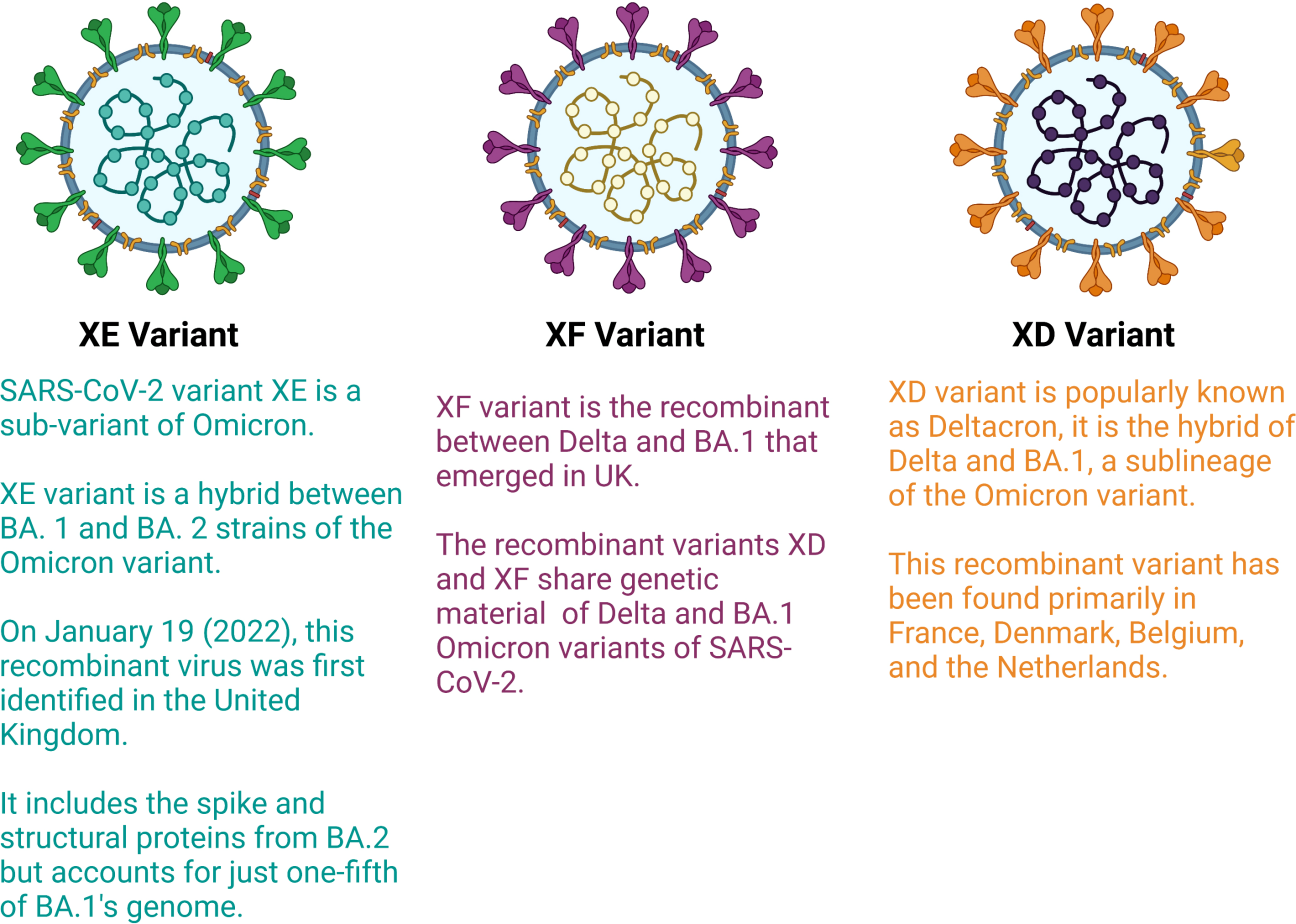
COVID-19 vaccine platforms used. (Some elements are created with BioRender.com).

The priority for vaccine development over anti-SARS-CoV-2 drug development is due to the intention to generate herd protection (49). With a worldwide effort, sharing of data, availability of large funding schemes and collaboration with industry, vaccines quickly became available and rolled out for emergency use. Both classic and novel platforms have been utilized (50, 51). Classic platforms used include virus-based vaccines (dead virus or live-attenuated virus) and protein-based vaccines; novel platforms include peptide, DNA, mRNA, viral vector and virus-like particle-based vaccines (52). Except for live-attenuated viral vaccines, all others require the presence of an adjuvant to enhance the antigenicity of the antigen (49, 53). The fact that most of the initial approved vaccines against SARS-CoV-2 that were rolled out were developed using novel platforms because these platforms are highly flexible and require less time for production than traditional methods (49, 54). Despite the novelty of the platforms deployed, the basic goal remains the same, i.e., the development of a safe and efficacious vaccine (**Figure 4**).

### Messenger ribonucleic acid-based vaccines

Messenger ribonucleic acid (mRNA) vaccines contain strands of desirable “transcripts of interest” inside a lipid nanoparticle (55). This coating protects mRNA against enzymatic degradation and aids entry into antigen-presenting cells in the lymph node near the site of injection. Once they enter the cell, mRNA is translated into protein and expressed as antigenic fragments on the surface to induce humoral (antibody) and cellular immunity (55–57).

The latest studies and technology breakthroughs (e.g., codon optimization, nucleotide alteration, and comprehending the significance of the 5’-untranslated region; UTR) have permitted mRNA to emerge as a viable therapeutic tool in the field of vaccine production by mitigating downsides such as instability and immunogenicity (55, 58). Currently, there are 45 mRNA vaccines in the clinical development phase, and 3 of these vaccines are approved for emergency use by various countries (59–61).

The use of mRNA has numerous beneficial features over other vaccine platforms. First, mRNA is a safe platform. It is non-infectious, as only part of the antigenic protein is produced. Because it does not penetrate the nucleus, there is no danger of insertional mutagenesis. In addition, it is destroyed using regular cellular activities, and the *in vivo* half-life may be controlled (58, 62, 63). Furthermore, the inherent antigenicity of mRNA may be reduced to alleviate the safety risk (58, 64, 65). The second distinguishing trait is its great effectiveness. During phase 3 studies, Spikevax (mRNA-1273) and Comirnaty (BNT162b2) showed effectiveness of 94.1% and 94.6%, respectively (66, 67). Extracellular RNases have a strong affinity for naked mRNA (68). As a result, lipid nanoparticles and the creation of modified nucleosides are required to enable mRNA entry into cells (57, 64, 65). Even though mRNA vaccines induce cellular as well as humoral immunity, multidose administration is required for effective protection. Importantly, no anti-vector immunity is generated. The third favorable feature is the scope of rapid, low-cost, and scalable manufacturing (56, 57, 69, 70). Last, it is easy to design and develop a new mRNA vaccine from an existing vaccine. Various antigens comprising the S1 subunit, the N-terminal region of the S protein and the receptor binding domain (RBD) have been used (71, 72). Nevertheless, it remains to be shown whether these antigen candidates are appropriate for inclusion in an mRNA vaccine to prevent COVID-19 in the long term.

mRNA-1273 was shown to stimulate T-helper (Th)-1 immune responses following the first dose, as 0.05% of circulatory CD4+ T cells were shown to secrete tumor necrosis factor (TNF)-alpha and/or interleukin (IL)-2 (73). After just one dose, both mRNA-1273 and BNT162b2 vaccinations generated levels of anti-RBD antibodies that were comparable to or higher than those seen in patients receiving convalescent plasma treatment However, CD8+ T-cell responses were induced only at modest levels. These findings show that protection following a single dose will have a very low neutralizing antibody (nAb) titre to provide sufficient protection against infection (74). Vaccines from Pfizer, Moderna, and AstraZeneca have all been shown to activate type I interferon (IFN), which could lead to pathogen-agnostic immunity (57, 75, 76).

Neutralizing antibody responses following one BNT162b2 immunization, in patients with cancer, was shown to be 67% which was further increased after the second vaccine dose. Spike protein-specific serum antibodies and T cells showed similar patterns in healthy controls, however, the magnitude was lower in cancer patients. Memory B-cell subsets were also noted to be potential predictors of anamnestic reactions to further immunizations in most cancer patients. Following these findings, a phase 1 trial of a third BNT162b2 injection was conducted in 20 participants with cancer (NCT04936997); the primary outcome was induction of immune responses. In sixteen subjects, a threefold increase in nAb responses one week following the third immunization were noted (77). However, T-cell responses remained unchanged following a third dose. In addition, sera of BNT162b2-vaccinated medical workers were found to efficiently neutralize the SARS-CoV-2 variation with the D614G substitution and the B.1.1.7 variant after the second dosage; however, the neutralization of the B.1.351 variant was decreased fivefold (78). Further, human breast milk contains humoral and cellular immune responses following mRNA injections. Antibodies to SARS-CoV-2 spike variants may be neutralized by milk anti-RBD antibodies. Nursed babies are given anti-RBD antibodies, in order to induce passive immunity and protection against SARS-CoV-2 (79). A booster dose with mRNA vaccine demonstrated better protection against the omicron variant (80). Furthermore, SARS-CoV-2-specific memory B-cell responses following the first dose of viral vector-based vaccines (ChAdOx1 nCov-19) may be stimulated by a booster with the mRNA-1273 vaccine (81). In a group of hospital workers who were administered two doses of either mRNA-1273 or BNT162b2 (82), vaccinations showed different effects on Fc-mediated effector functions and epitope-specific antibody responses. Depletion of RBD-specific antibodies emphasized these variations even further. These findings implied that mRNA-1273 and BNT162b2 induce different immune responses despite having identical chemical compositions. The third (booster) dose of BNT162b2 has been noted to reduce the number of confirmed infections, severity of sickness, hospitalisations, and death against several VOCs.

### Inactivated vaccines

A conventional technique of inactivating the whole pathogen with physical (thermal stimuli, ultraviolet light) and/or chemical treatment followed by a purification process is a proven tool that has been deployed for decades for effective protection against a wide range of diseases, such as, polio, rabies, pertussis, influenza (83, 84) Inactivated viruses cannot produce or replicate and thus, do not produce any pathological complications but are still capable of producing an immunogenic response. Inactivated vaccines show little or no effect on cell-mediated immunity (84, 85). Thus, the effective immune response develops only after the second and/or booster dose. Favorable features of this platform include a high level of safety, reasonable efficacy, and ease of large-scale production (86–88). Additionally, logistical challenges are less stringent for such vaccines. However, inactivated pathogens are always formulated with the addition of immune potentiators (e.g., aluminum preparations) (84, 89). Several precautions in manufacturing (Bio-Safety Level 3) need to be followed. Currently, there are 21 inactive vaccines in the clinical development phase (59–61). CoronaVac and covaxin have shown 50-81% efficacy in several studies (85, 90). Covaxin has also been reported to induce nAbs against the alpha variant (90, 91). A prospective observational study among covaxin recipients was carried out in a tertiary care facility in India between June 28 and September 6, 2021. The study reported at least one adverse event following immunization (AEFI) in 29.8% of individuals. There were no severe adverse events reported, and 1.6% suffered moderate AEFI. The individuals frequently reported myalgia (59%) and pain at the injection site (14.6%, 9.7%). When compared with the second dose (26.4%), the incidence of AEFI after the initial dose was higher (38.1%). Additionally, the study concluded that female sex, history of an allergic reaction, presence of comorbidities, acute infection in the past three months, and use of chronic drugs were the main risk factors for AEFI (92).

### Live-attenuated vaccines

Live-attenuated vaccines contain a variety of living pathogens that have been weakened under laboratory conditions (93). Live-attenuated vaccines are developed by techniques such as repeated subculturing and codon pair deoptimisation (94). MMR (mumps, measles, and rubella), polio, and chickenpox vaccines are some examples that have been used for decades. The more resemblance to the original pathogen, the better the immune response generated. Live-attenuated viruses are capable of replicating themselves within cells, but their virulence power is reduced (93, 95). Because of the capability of replication within cells, live-attenuated vaccines have the ability to generate strong cell-mediated immunity in addition to humoral immunity, which is desirable in viral infections (96). Continual antigenic stimuli produced by live attenuated vaccines are sufficient to induce memory cells. They are less safe than inactivated vaccines, as the weakened pathogen can rarely revert to its original wild-type form and can lead to serious adverse events or disease itself (95, 96). Care during cold storage and reconstitution (in the case of lyophilised vaccines) must be taken; otherwise, potential immunization errors can occur (93). A series of live-attenuated vaccine candidates for SARS_CoV-1 (known as SARS) and Middle Eastern respiratory syndrome (MERS, MERS-CoV) were developed and showed good immunogenic responses (97–102). Despite the huge potential of live-attenuated vaccines in terms of generating strong immunogenic responses, only 2 candidates are currently in clinical trials (59). COVI-VAC is one such example.

### Protein subunit vaccines

Protein subunit vaccines contain purified small antigenic proteins instead of the whole pathogen. These fragments are usually proteins but can also be polysaccharides (as in the case of some bacterial vaccines). In order to generate strong immune responses, polysaccharide fractions are usually conjugated with proteins. Protein-based vaccines are considered safe and contain purified fragments that are not capable of producing disease. However, they are less capable of inducing long-lasting immunogenic responses. Their limited ability to generate optimal cellular responses can be enhanced by the addition of immune potentiators and the use of novel delivery systems (103–105). Some protein-based vaccines also require booster doses to produce effective protection against the pathogen. Hepatitis B, pneumococcal, and pertussis vaccines are some examples of protein-based vaccines.

The domains of the S protein (both S1 and S2 subunits) of the SARS-CoV-2 virus are considered important for hACE2 receptor-mediated endocytosis for entry into human cells (104, 106). Therefore, the administration of purified S protein of SARS-CoV-2 and its fractional RBD for the development of nAbs and cellular immunity was thoroughly studied (107). NVX-CoV2373, SCB-2019, and ZF2001 vaccines are some examples being developed using this approach (108, 109). Another potential target, the N protein, is expressed in a stable and abundant amount during SARS-CoV-2 infection and is highly immunogenic (110). Coexpression of the recombinant spike, membrane, and envelope proteins have been explored (111). There are sixty-nine candidate vaccines, of which thirty-two are in phase 3 human clinical trials (59–61). One example is NVX-CoV2373 (known as Novavax; sold under the name of Nuvaxovid and Covovax), which showed 86.3% efficacy against alpha variant and 96.4% against other non-alpha variants (112). In another phase 3 study, NVX-CoV2373 showed some local effects such as tenderness, pain, fatigue with low medically attended adverse events and serious adverse events and no episodes of anaphylaxis. When combined with flu vaccine, the efficacy of the NVX-CoV2373 vaccine decreased from 89.8 in NVX-CoV2373 alone group compared to 87.5% efficacy when combined with flu vaccine (112). The NVX-CoV2373 vaccine, displayed cross-reactive immune responses against omicron (B.1.1.529) and other variants following a two-dose primary regimen. After the third dose, immunological responses were comparable to or beyond levels associated with protection in phase 3 clinical trials, with a 9.3-fold increase in IgG and a 19.9-fold increase in ACE2 inhibition. Immune responses in adolescents were 2- to 4-fold stronger than those of adults against a wide range of variants (113).

### Viral vector-based vaccines

Vaccines generally use viral antigenic material either naturally or synthetically to induce an immune response; however, the process varies with different vaccine platforms (114). Viral vector-based vaccines, on the other hand, use a viral vector that encodes the gene for the viral-specific antigen, and once injected, they use host cell machinery to produce such viral antigen (114, 115). The vaccine simulates the pathology of natural infection with some pathogens, specifically viruses, by contaminating cells and imposing them to produce large amounts of the antigen, following activation of an immune response (both T-cell- and B-cell-mediated) (116). To produce effective vaccines quickly against various infectious diseases, the adenoviral vector has been favored. As of 2 June 2022, there were 29 viral vector vaccines under development, and 24 were adenovirus-based vaccines (10 in phase 3, 5 in phase 2, and 9 in phase 1) (117). To date, 6 viral vector vaccines have been approved under the emergency use authorization pathway in different countries and 3 vaccines, Ad26.COV2.S, Vaxzervria, and Covishield™ (Oxford/AstraZeneca formulation) have been granted emergency use listing by the WHO.

Efficacy data of all vaccine candidates currently in phase 3 and 4 are reported to be more than satisfactory. However, the Oxford-AstraZeneca vaccines, sold under the brand names of Vaxzervria and Covishield™ which uses a modified chimpanzee adenovirus vector ChAdOx1 were noted to cause rare but life-threatening thromboembolic adverse events in some patients. These adverse events involve the generation of an antibodies which cross react with platelet factor 4, thus initiating a clotting cascade (118). Presently, the EMA and WHO have clarified that the risk of developing thromboembolic events is not significantly higher than that in the general population (119). One should understand that as of now, the benefits outweigh the risk associated with the use of such vaccines. Nevertheless, long-term evaluation studies are required. A single dose of ChAdOx1 vaccine was demonstrated to evoke polyfunctional antibodies capable of affecting neutralization as well as a variety of additional antibody-dependent effector mechanisms, all of which may aid in disease prevention. Antibodies generated by ChAdOx1 were found to aid phagocytosis and were capable of antibody-depended complement deposition after only one dose which were further boosted after the second dose (120). Furthermore, because of the stimulation of TNF and IFN by CD4+ T cells in response to antigen stimulation *in vitro*, this vaccine produced robust T-cell responses that peaked 14 days after a single dose. Despite decreased T-cell responses and greater antibody responses after the second dosage, the vaccine’s effectiveness following one and two doses is identical, suggesting that alternative protective mechanisms are active after one versus two doses. Prolonging the interval between doses also increased immunogenicity and efficacy (121).

The Janssen vaccine sold under the brand name Jcovden is produced by Janssen vaccines in Netherlands and Belgium and by its subsidiary USA company Johnson & Johnson. This vaccine is a viral vector based human adenovirus vaccine (Ad26.COV2.S) which contains the spike protein. The vaccine is currently approved for emergency use in 111 countries, while the global phase 3 clinical trial is ongoing (NCT04505722 and NCT04614948). In a research study conducted during the delta variant pandemic in hospitalized individuals, the vaccine was found to be 81% effective (122). This cohort study in US clinical practice demonstrated consistent vaccine efficacy of Ad26.COV2.S for at least six months prior to and throughout the emergence and dominance of the delta variant. Similarly, the Sisonke single-arm, open-label, phase 3B study conducted in South Africa, demonstrated efficiency of a single-dose of Ad26.COV2.S vaccine against severe COVID-19 illness and COVID-19-related mortality; as well as against both beta and delta variants, and provided empirical support for its widespread usage (123). A single dose of Ad26.COV2.S offered 83% protection against COVID-19-related deaths, 75% against hospitalisations requiring critical or intensive care, and 67% protection against COVID-19-related hospitalisations (123). The effectiveness was also shown in older health-care workers and participants with comorbid HIV infection (123). Similar efficacy was noted against COVID-related hospitalisations and deaths during the beta and delta waves. Protection persisted for at least 6 months (NCT04505722). Despite the efficacy of inducing immunity with only one injection and efficacy in preventing hospitalisations and deaths, cases of thrombocytopenia and Guillain Barre syndrome have been reported. In February 2022, Johnson& Johnson announced that it halted production of Ad26.COV2.S and would resume at some point in the future.

### Virus-like particles

Virus-like particles are adaptable, safe, and are able to stimulate strong humoral and cellular immune responses. They consist of repeated viral surface proteins, are small (20-200 nm), do not replicate and are alternatives to attenuated virus-based vaccines. The currently approved hepatitis-B and human papillomavirus vaccines use this technology. The CoVLP+AS03 (brand name Covifenz) vaccine developed by Medicago in Canada and by GalxoSmithKline comprises virus-like particles grown in *Nicotiana benthamiana* weed. These virus-like particles are made using a molecular farming technology and as such, it is low-cost, safe and easy and quick to produce. CoVLP+AS03 adjuvant was shown to be effective in preventing COVID-19 caused by a spectrum of variants, with vaccine efficacy ranging from 69.5% against symptomatic infection to 78.8% against moderate-to-severe disease (124). In addition, CoVLP vaccine exhibited 100% efficacy against the alpha variant, 75.3% against delta, and 88.6% against the gamma variant.

### Comparison of vaccinations

“SARS-CoV-2-spike-specific immune responses to Moderna mRNA-1273, Pfizer/BioNTech BNT162b2, Janssen Ad26.COV2. S, and Novavax NVX-CoV2373 were studied longitudinally for 6 months” by Zeli Zhang and colleagues. After mRNA or NVX-CoV2373 immunization, 100% of people developed memory CD4+ T cells, and CD4-CD8 was overrepresented. Although memory CD8+ T cells were identifiable in only 60-67% of subjects at 6 months, mRNA vaccinations and Ad26.COV2. S generated equivalent CD8+ T-cell frequencies. As per the study, although Ad26.COV2. S T cell, B cell, and antibody responses were relatively constant over 6 months, it was not the strongest immunogen by any metric. A high frequency of CXCR3+ memory B cells was a distinguishing feature of Ad26.COV2 vaccination. Over 6 months, neutralizing antibodies in mRNA vaccines decreased significantly, whereas memory T cells and B cells remained rather steady (125).

In healthy adults who received the full dosage of inactivated vaccine (CoronaVac, also known as Sinovac), the booster effect of multiple vaccination platforms, including inactivated vaccine (BBIBP), viral vector vaccine (AZD122), and mRNA vaccine (BNT162b2) were assessed. The outcome demonstrated that the booster dose was safe and had no negative side effects. Additionally, the immunogenicity demonstrated that the booster dose of the viral vector and mRNA vaccine resulted in a significant quantity of Ig anti-RBD, IgG anti-RBD, and IgA anti-S1 booster responses. In contrast, the booster response for inactivated vaccination was lower than for other vaccines. Therefore, against wild-type SARS-CoV-2 and its variants (B.1.1.7-alpha, B.1.351-beta, and B.1.617.2-delta), the neutralizing activity of vaccine serum had a strong inhibition of over 90%. Additionally, IgG anti-nucleocapsid was only detected in the BBIBP booster group. After receiving the additional viral vector or mRNA booster vaccine, the study showed a marked rise in the levels of the IFN-secreting T-cell response (126). Between January 1 and August 3, 2021, another retrospective cohort study involving healthcare workers (HCWs) was carried out in Brazil. A total of 13,813 HCWs were included in the study: 46.2% of them received the CoronaVac vaccine, 42.8% received the ChAdOx1 vaccine, and 11.0% were not immunized. In all, COVID-19 infection cases occurred in 6% of HCWs who were not vaccinated, 3% of HCWs who received two doses of the CoronaVac vaccine, and 0.7% of HCWs who received two doses of the ChAdOx1 vaccine. The estimated vaccination efficacy for ChAdOx1 and CoronaVac was 88.1 and 51.3%, respectively, in the adjusted analyzes. The need for mechanical breathing, hospitalisations, and length of stay were all decreased by both immunizations. For mutations of interest, SARS-CoV-2 samples from 19 HCWs were evaluated. Eighteen of the samples contained the gamma SARS-CoV-2 mutation (127).

**Figure 5:**
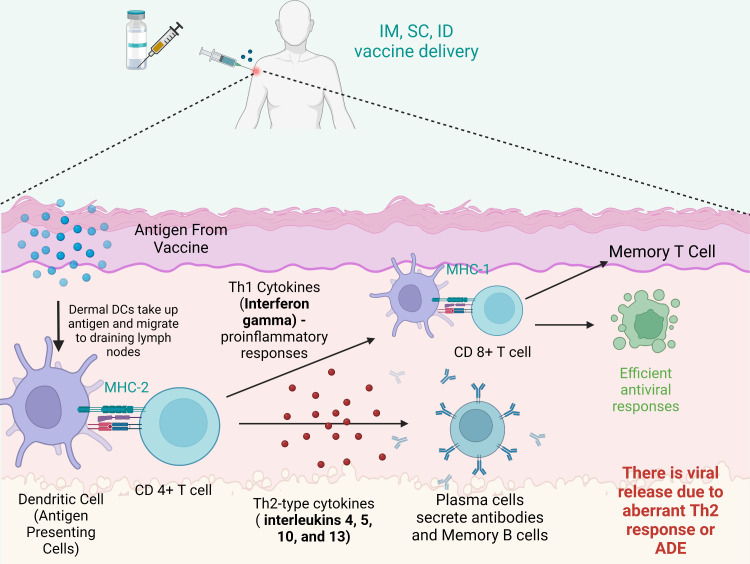
Immune reaction after vaccination. After intramuscular (IM), intradermal (ID) or subcutaneous (SC) vaccine delivery, dermal dendritic cells (DCs) take up antigens and migrate to draining lymph nodes to stimulate T cells (CD8+ T cells and CD4+ T cells). Plasma cells secrete antibodies and memory B cells. CD8+ T cells can be stimulated by Th1 cytokines and in turn acquire the ability to attack the infected cells. However, imbalanced immune responses have the potential to cause pulmonary immunopathology, partially due to an aberrant Th2 response or antibody-dependent enhancement (ADE). Created with BioRender.com.

Considering the current emerging variants of SARS-CoV-2 and the efficacy of the currently available vaccines, it is clear that one needs variant-specific vaccines rather than repeated booster dosing with the existing vaccine, as each vaccine under an emergency use approval license has some degree of adverse events reported, and none provide complete protection against the emerging variants. **Figure 5** summarizes the immune responses post vaccination.

## Impact of variants on vaccine efficacy

The twist created by the spread of mutant strains has put a question mark on the efficacy of the current COVID-19 vaccines, although some of these vaccine candidates have also proven their efficacy against some of these mutant strains. The emergence of postvaccine reinfections with SARS-CoV-2 lineages can have a substantial influence on public health and the economy. The possible use of regular vaccination shots at 6 months or 1 year for the control of variants cannot be ruled out. As of now, this disease has been very unpredictable, and it is still too early for such assumptions. Logistic challenges associated with nucleic acid-based and vector-based vaccines, especially in poor and developing countries, are still a concern. The SARS-CoV-2 pandemic has shown many unpredictable outcomes, but the scientific community is optimistic in the fight against all challenges.

Researchers are competing against time to build immunity against this intriguing virus, whose capacity to change and evolve appears to be exceeding our capability to attain herd immunity. The long-term COVID-19 pandemic has been dependent on balance by a dispersal of approved vaccines and emerging viral variants (22, 128). Due to new variants, the race to the finish line may be a sprint. The variants are worrisome for several reasons. First, the VOCs transmit at least 20-50% faster from person to person. This encourages them to infect more individuals and spread more quickly and widely, eventually becoming the prevailing paradigm. Second, there is a concern that SARS-CoV-2 mutations can result in them being more contagious, induce more severe sickness, as well as an increase in hospitalisations and mortality (**Table 1**) (129). Although vaccine effectiveness is necessary for vaccine licensure, it may not always represent the impact of immunization in the actual world, particularly when clinical trials for vaccines involve primarily younger, healthy individuals rather than those most at risk of extreme infection, and these studies took place before some of the more recently reported SARS-CoV-2 variants were discovered (130). As a consequence, it is crucial to determine the degree and duration of defense against infection or illness across all age groups and populations; in the case of COVID-19, this is especially critical given the greater risk of severe disease in older adults (131). The delta variant has the same high resistance as other omicron sub-variants and no increased sensitivity to serum acquired during the delta wave (132). Even though the impact of delta-derived spike mutations in the N-terminal region on viral proliferation and pathogenicity is unknown, they do not seem to decrease neutralization resistance. Recombination of viral variants and the possibility of the formation of a far more virulent variety with significant immune evasion is a major issue that must be closely monitored.

The waning of protection has been seen with time since vaccination, particularly with the delta variant, which is able to at least to some extent evade natural and vaccine-induced immunity. However, booster doses provide a rapid and significant boost in protection against both mild and severe diseases (34, 133). The epidemiology of viral VOCs along with the efficacy of EUA vaccines is vital for creating immunization strategies and the advancement of novel vaccines (**Table 2**). In contrast to similar BNT162b2 vaccination, heterologous BNT162b2 boost following ChAdOx1 nCoV-19 reliably results in greater neutralizing titres against the alpha, beta and gamma variants. Surprisingly, homologous BNT162b2 prime boost appears to be more effective in producing delta variant-neutralizing antibodies (150, 151). Furthermore, there is presently a scarcity of data on the duration of protection provided by mRNA vaccines. There are currently no effective vaccination measures or conclusive empirical estimates of vaccine longevity. Due to decreased nAbs, reinfection with different variants of SARS-CoV-2 has been observed. The COVID-19 vaccination booster dosage is critical for the production of neutralizing immune reactions against omicron, according to new findings, and improved therapeutic antibodies are required for this and future versions (152). A recent study revealed that natural infection significantly increases the number, quality, and diversity of humoral immunity, irrespective of whether it happens before or after vaccination (153). Hybrid immunity, which is the result of prior COVID-19 and a subsequent vaccine, seems to provide the best protection against SARS-CoV-2 infections; however, there are still significant information limitations in this area.

**Table 2:** Coronavirus variants and effectiveness of the current vaccines.

Vaccine candidate	% Efficacy reported during Phase 3 trial	Specific Comments regarding effectiveness	Effectiveness against variants						Reference
			Alpha	Beta	Gamma	Delta	Epsilon	Omicron	
Comirnaty (BNT162b2)	94.6%	• This vaccine’s efficacy against the Delta infection peaked at 68% (95%CI: 64-71%) and 62% (95%CI: 57-66%). • After taking one dosage, neither age group is protected against Omicron infection. • The vaccine proved efficient against virus strains; however, vaccine potency against severe and mild infection after two doses is less for the Omicron variant than for the Delta variant, and fading is quicker.	Yes, 95% (CI): 1.2–2.1)	Yes, (95% CI: 6.4–14.4)	Yes, (95% CI: 1.6–3)	Yes, at 68% (95%CI: 64-71%) and 62% (95%CI: 57-66%).	Yes	No, one dosage, neither age group is protected	(134–138)
Spikevax (m-RNA-1273)	94.1%	• One dosage of mRNA-1273 had lesser protection against all variants than several doses, with protection against mu at 45.8% (0.0% to 88.9%) and alpha at 90.1% (82.9% to 94.2%). • Vaccine efficiency against delta variant infection was best for 14-60 days (94.1% (90.5% to 96.3%)) and fell substantially, with vaccine effectiveness of 80.0% (70.2% to 86.6%) at 151-180 days and with nondelta variants similarly declined for delta (from 98.6% (97.3% to 99.3%) at 14-60 days to 88.7% (73.2% to 95.2%) at 121-150 days). • At 14–60 days, vaccine efficacy against unknown variants was 83.6% (79.5%–86.9%), and it decreased to 68.5% (51.3%–79.6%) at 151–180 days.	Yes, 90.1% (82.9% to 94.2%)	Yes, leaser effective	Yes, lesser effective	Yes, 14-60 days (94.1% (90.5% to 96.3%) 80.0% (70.2% to 86.6%) at 151-180 days 88.7% (73.2% to 95.2%) at 121-150 days).	No	No	(139, 140)
Vaxzevria and Covishield	–	• Vaxzevria was efficient but because data was only provided after a single dosage rather than the recommended two dose schedule where potency is boosted. • The vaccine was 50% protective against the Beta/Gamma mutations and 70% and 72% effective against the Delta and Alpha variants, respectively.	Yes, 72%	Yes, 50%	Yes, 50%	Yes, 70%	No	No	(141, 142)
Sputnik V	91%	• The studies show that Sputnik V counteracts the Omicron variant by producing a strong antibody response. • Among the top quartile of those with strong RBD-specific IgG antibodies, 100% of those vaccinated with Sputnik V were able to neutralize the Omicron variant, compared to 83.3% of those immunised with Pfizer. • Comparatively, 56.9% of those who received the Pfizer vaccine were able to neutralize Omicron, compared to 74.2% of those who received the Sputnik V vaccine.	Yes, 85.7% (95% CI 84.3–86.9%) and 97.5% (95% CI 95.6–98.6%	Yes, 80%	No	Yes, Effectiveness: 87.6% (60–79-year-old), 75.28%(up to 60 year),	Yes	Yes, neutralize with strong RBD -specific IgG antibodies, 83.3% more efficacy than Pfizer,	(143)
Sputnik light	79.4%	• Using Sputnik Light as a booster enhances the virus’s ability to neutralize the Omicron variant. • It has a 70% success rate against the Delta variant.	Yes, less effective	No	Yes, 70%	Yes, effectiveness: 88.61%, (18-29-year-old group), 88.61%(88.61%)	No	Yes, Neutralize variants	(144, 145)
COVID-19 Vaccine Janssen (JNJ-78436735; Ad26.COV2.S)	85%	• This vaccine was proven to be effective in clinical trials against multiple variants, especially B1.351 and P.2. • Data on how well this vaccine works against the Omicron version are yet lacking.	No	Yes, more effective	Yes, more effective	No	No	Yes, lesser efficacy	(134–136, 146)
CoronaVac	51% against symptomatic cases 100% against hospitalized patients	• The estimated efficiency of Sinovac-CoronaVac among health professionals in Manaus, Brazil, was determined in a survey study, although there is currently inadequate data for Omicron.	Yes, 53–66%	No	Yes, 51% to 84%	Yes, 91%-93%	No	No	(147)
BBIBP-CorV	78.1% against symptomatic cases 100% against severe cases	• The efficacy of the BBIBP-CorV vaccine in severe cases was 80%, 92%, and 97% against hospitalisation, critical care admission, and death, respectively.	No	Yes, neutralizing antibody responses	No	Yes, neutralizing antibody responses	No	No	(148)
Covaxin (BBV152)	77.8%	• The effectiveness of the vaccine against all variant-related COVID-19 diseases was 71%, with efficacy against Kappa and Delta being 90% and 65%, respectively. • If additional VOCs emerge that impact vaccination performance, these guidelines will be revised. • There is currently no information available for Omicron.	Yes, 71%	Yes, 71%	Yes, 71%	Yes, 65%	Yes, 71%	No	(22, 149)

## Vaccines: Did they save the world?

There is no doubt that mass vaccinations have revolutionized global health. The single most innovative medical outcome was the eradication of smallpox following worldwide mass vaccinations, and more recently, on 28 August 2020, polio was declared eradicated in Africa following a mass vaccination campaign that started in 1980. Likewise, a huge decrease in the number of incidences and mortalities against measles, mumps, rubella, chickenpox, whooping cough, tuberculosis, etc., have been recorded in the last few decades with childhood vaccination programs. Within the first year of COVID-19 vaccine rollouts, it is estimated to have saved 19.8 million lives, and thus far, over 12 billion doses of COVID-19 vaccines have been administered in most countries around the world. The aim of the current vaccines was to reduce severe symptoms, illness, hospitalisations and death (154). Indeed, COVID-19 vaccines have saved millions of lives thus far, even though breakthroughs are common. COVID-19 continues to be a pandemic, with thousands of daily cases and hundreds of daily deaths, and as the virus continues to circulate, new variants emerge, some of which are more transmissive and more dangerous, including some variants that reduce the efficacy of vaccines (154, 155). Further investments and the development of new improved vaccines against the variants are required and should be administered annually, similar to influenza virus vaccines (155).

## Risks associated with fast-track vaccine evaluation

There is a race to develop a vaccine that is both safe and effective against COVID-19. With a global effort and collaboration and funding, one could be in the market within 12–18 months. One fully approved vaccine and 38 emergency use approved (EUA) vaccines for COVID-19 in the market in less than two years define the speed of the vaccine development race. Because of the expedited pace of innovation, global health researchers are warning that vaccines will be authorized with insufficient data and analysis. For instance, at least one candidate did not undergo animal testing. In the meantime, CanSino Biologics’ exploratory COVID-19 vaccine was authorized for use in the Chinese military before the completion of phase 3 trials (156). One of the deepest concerns is the possibility that a fast-tracked vaccine will have unexpected effects. No vaccine is completely safe, but if a billion people are vaccinated, one in 10,000 major negative incidents will impact 100,000 of those people. In May 2021, it was demonstrated that four of the 45 participants in Moderna’s phase 1 vaccine trial had medically significant adverse events (157). In identifying the severity of the existing public health crisis and the idea of making a vaccine accessible as quickly as possible, one assumes that a median 2-month follow-up after completion of the vaccine regimen will provide the necessary safety and efficacy data to endorse the allocation of an experimental vaccine under emergency use authorization (158).

Phase 3 randomized trials that investigate the incidence of COVID-19 in large groups of immunised and nonimmunised patients are the actual test of the vaccine’s efficacy. This type of study will determine whether one, many, or none of the new COVID-19 vaccines offer effective or limited immunity and whether their usage is associated with serious side effects. Science-based initiatives and significant funding have supported the development of 38 vaccine candidates that will be licensed for public use and contend head-to-head with each other (159).

**Figure 6:**
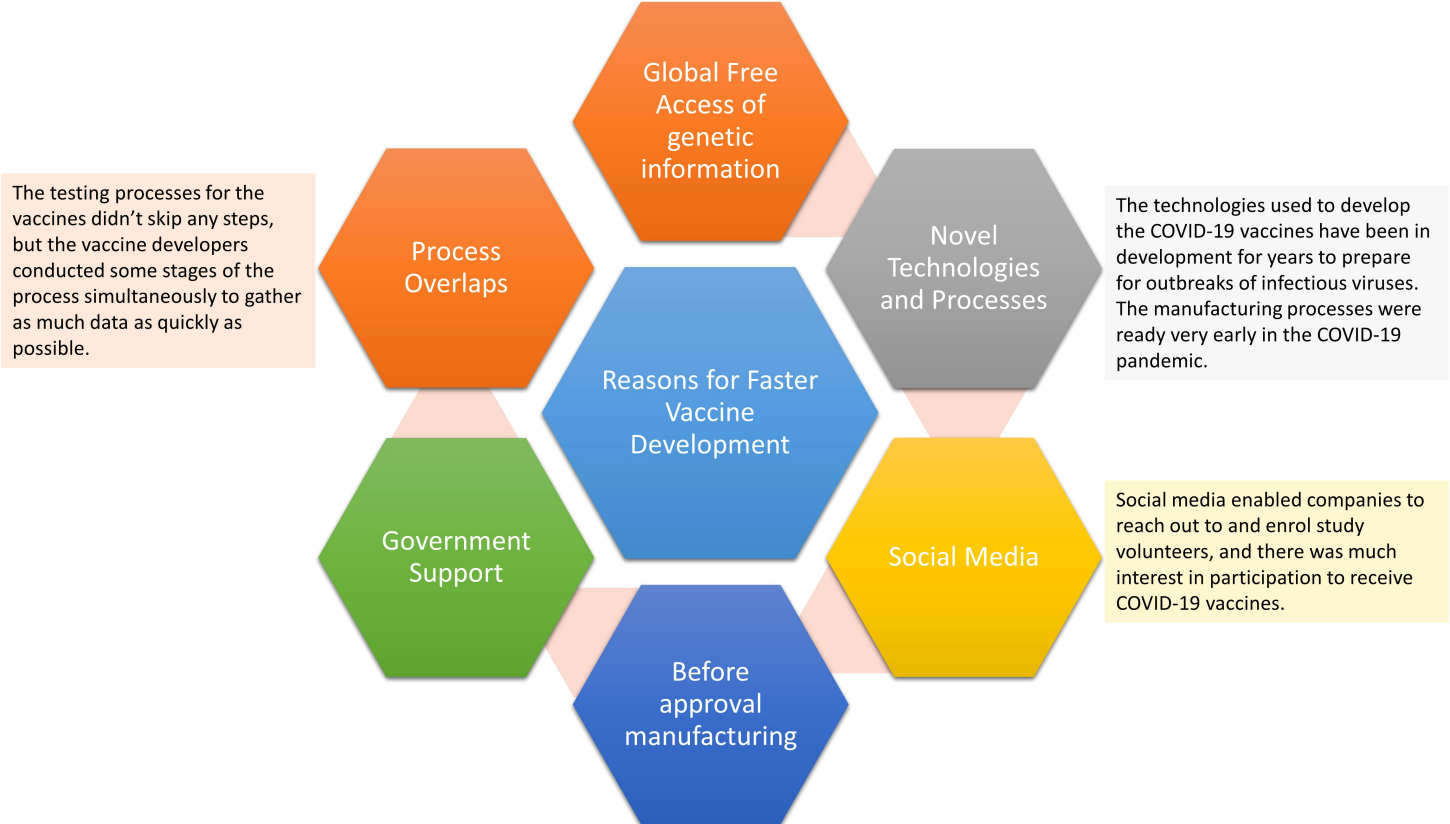
Reasons for fast-track vaccines for COVID-19.

The most important consideration is that vaccines against SARS-CoV-2 need to be dosed multiple times in the world population in a timely manner, at a level far beyond other types of vaccinations in history. The global vaccine supply chain has played the most crucial role in delivering each vaccine shot to end users (160). In the last two years, the government and healthcare organizations worldwide have faced the complex task of obtaining and distributing supplies to their populations and administering the vaccine (**Figure 6**). While a handful of countries and companies managed to receive approval for vaccines with the required efficacy, acquiring sufficient doses for all the countries is just the start. Vaccines must then be transported securely to multiple locations and preserved at the right temperature as well as be protected from tampering to assure product integrity. While all types of vaccines must be transported in cold conditions, creating secure cold chain management of the global vaccine chain supply is vital (161). This global pandemic shows how complex global supply chains developed to deliver billions of vaccine doses. Global COVID-19 vaccine supply chains facilitated sustained production and access. Even though obtaining enough vaccines remained difficult for many countries last year, the worldwide trade of vaccines was 26% higher during the first six months of 2021 compared with the full 12 months of 2020. The vaccine business was also supplemented by increasing trade in associated or intermediate inputs, strengthening their wider manufacturing and distribution. Even through nationalistic policies from the United States and European Union, international interdependence still emerged. This highlights the capacity of global supply chains to ramp up and boost the distribution and production of essential vaccines in record time. A global supply chain is still important in immunizing all parts of the world to lower the risk of new variants emerging.

## Ethical issues

**Figure 7:**
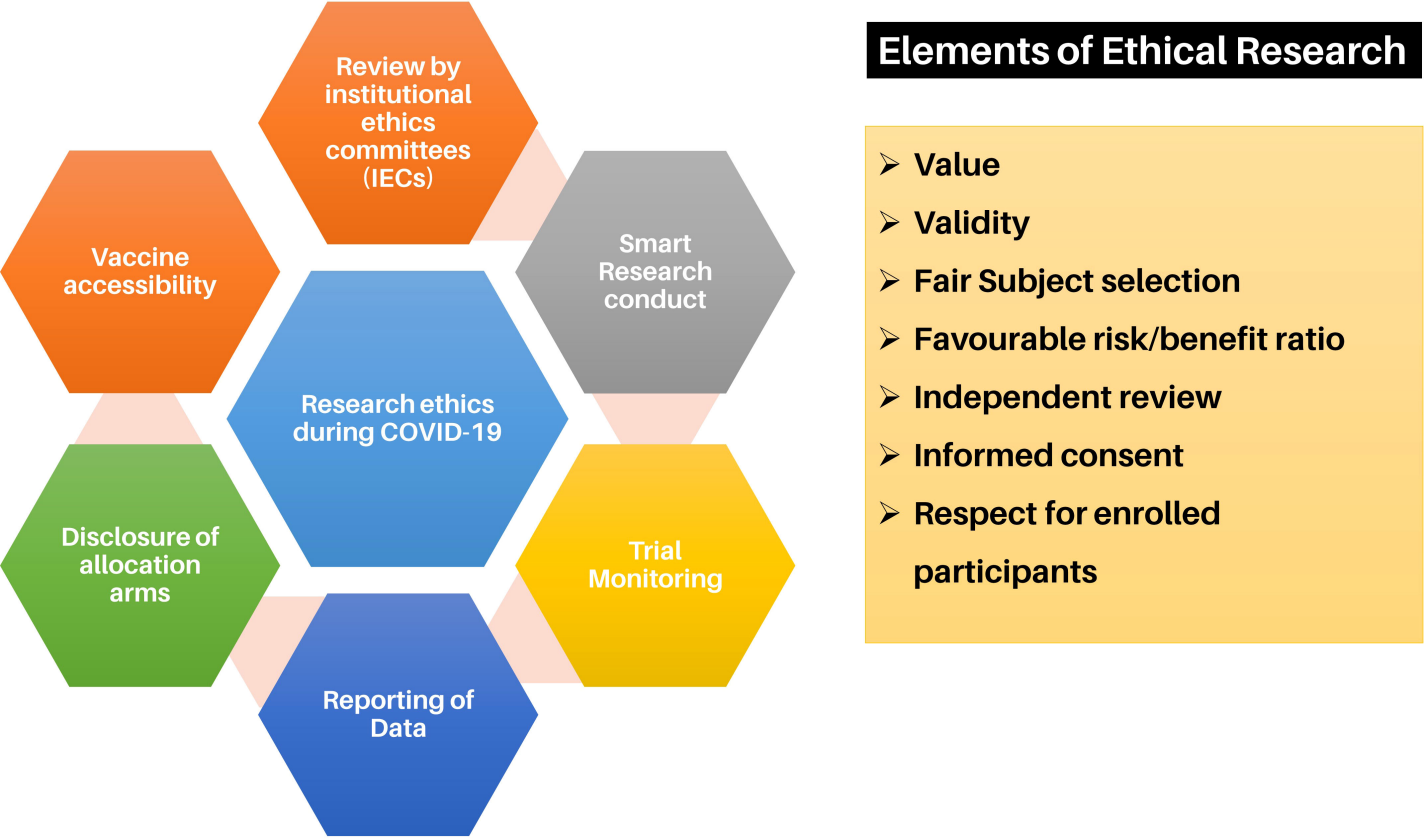
Ethical considerations for vaccine research for COVID-19 (162, 163).

Ethical considerations to develop a vaccine and be tested in humans must be met, and approvals must be in place. There are standard procedures regarding clinical trials and testing of vaccines in humans (**Figure 7**). From preclinical *in vitro* and *in vivo* studies to larger animal immunological and toxicology studies to human phase 1, 2, 4 clinical trials, strict ethical guidelines and procedures must be met, and the obligation of reporting human clinical outcomes follows a clear path (164). However, several other ethical issues regarding vaccination against SARS-CoV-2 attracted much controversy at every stage of its development and use. Vaccines were developed in record time and as such raised many issues with the population, and it was difficult to persuade some reluctant populations to receive the vaccine. In addition, government mandates were an issue with some that raised much debate regarding their moral rights vs a duty. However, the ethical debate was not only to those who refused to be vaccinated but also to those who rushed or jumped queues to be first. Several ethical issues have had to be overcome regarding which population group should be the first to receive the vaccine and what could be done with those who were reluctant. In addition, there were issues regarding which countries and groups should benefit before others. As such, some countries, i.e., the US, Europe and Australia mandated the vaccine to reach over an 80-90% vaccination rate, while leaving out other nations generally low-income nations. Vaccine passports also raised issues to an unequally vaccinated world, where those who were vaccinated were able to go to work, go to shops and restaurants, engage in leisure activities and travel (165). Overall, the inadequate immunization coverage, even though the vaccines were shown to be safe and effective, at least in the time frame tested, the uncontrolled number of COVID-19 cases and hospitalisations, and the rapid upsurge of more new variants tipped the scales towards some form of mandatory vaccination policy (39, 166). Voluntary vaccination compared to compulsory vaccination policies is at the center of debate in the last 2 years.

## Future outlook

COVID-19 shows that rising infectious diseases present a substantial and growing danger to global health, the human and economic costs are vast, and many other viruses could result in another pandemic. To minimize pathogenicity in humans, entire pathogens were initially utilized as vaccine agents, either inactivated or attenuated. Various mRNA- and DNA-based vaccines were authorized for the first time in human history. The development of vaccine formulations against COVID-19 within record time in this pandemic by following the same protocols for previously emerging SARS-CoV and MERS-CoV and as an emergency vaccination discovery approach to skip over some preliminary preclinical steps of vaccine development has allowed for the creation of the life-saving shots for the eight billion global population in two years. Unexpected correlations between parameters were discovered during the deconvolution of patient features inside groups. By combining large datasets using uniform manifold approximation and projection-assisted clustering, patient groupings containing precise estimates may be identified, as well as unanticipated relationships among clinical factors. This use of machine learning to delineate illness pathophysiology and possible treatment strategies is a strong strategy (167).

Vaccine effectiveness, in contrast to vaccine efficacy, refers to the reduced risk of illness or sickness among vaccinated people. According to Zhang and colleagues (125), “head-to-head comparisons of T cell, B cell, and antibody responses to diverse vaccines in humans are likely to be informative for understanding protective immunity against COVID-19, with particular interest in immune memory. Here, SARS-CoV-2-spike-specific immune responses to Moderna mRNA-1273, Pfizer/BioNTech BNT162b2, Janssen Ad26.COV2. S, and Novavax NVX-CoV2373 were examined longitudinally for 6 months. One hundred percent of individuals made memory CD4+ T cells, with SARS-CoV-2 spike-specific circulating follicular helper T cells (cTfhs) and CD4-CTLs highly represented after mRNA or NVX-CoV2373 vaccination. mRNA vaccines and Ad26.COV2. S induced comparable CD8+ T-cell frequencies, although only detectable in 60-67% of subjects at 6 months. A differentiating feature of Ad26.COV2. S immunization resulted in a high frequency of CXCR3+ memory B cells. mRNA vaccines had substantial declines in antibodies, while memory T and B cells were comparatively stable. These results may also be relevant for insights against other pathogens.” The vaccine’s population-dependent effects, along with immunization schedules and vaccine processing, can have an impact on this. Various large-scale research studies have established why it is essential to have vaccinations, as they offer a considerably greater level of protection against COVID-19, including against new variants of concern, whether you have been previously infected or not. However, the analysis also evidently reveals that this protection from just two primary doses vanishes significantly within months, which is why the rollout of booster shots has been effective to avoid infection and illness, particularly against new COVID-19 variants. Socioeconomic COVID-19 risks of the pandemic and sociomedical safety measures as a means of flattening the pandemic curve and preserving vaccine supplies as well as integrated vaccination therapeutic tactics to tackle the COVID-19 pandemic. Spike amino acid replacements and subtractions that affect neutralizing antibodies are common in the worldwide virus demography, and research suggests that the variants exhibit resistance to antibody-mediated immunity induced by vaccines. Attempts are ongoing to stop the spread of the virus from the local site by IgA-mediated protection with the help of intranasal route of administration and DC-based vaccines (168–170).

## Conclusion

In the middle of the high death rate and the alarming fall of the global healthcare system in the initial two years of the COVID-19 pandemic, SARS-CoV-2 vaccines were approved for the first time based on novel vaccine technologies (either RNA or DNA). Fast-track vaccine approval by regulators, manufacturing of vaccines at a large scale, and the effective distribution of COVID-19 vaccines have played a significant role in distributing vaccine shots that provide robust protection against infectious SARS-CoV-2 and its evolving variants. Fortunately, even for those who become infected, vaccination protects against major disease, hospitalisation, and fatality from COVID-19. The key vaccine development processes and vaccination programs are still ongoing because of the devastating effects of the pandemic. Even the most industrially developed and economically prosperous nations can succumb to the pandemic. Therefore, new manufacturing facilities, steadfast cold-chain supply chain networks, and continuous research should be in place to streamline development and maintain the safety and efficacy of future vaccines. Unparalleled as the COVID-19 pandemic might have been, more variants of SARS-CoV-2 will enter the circulation, and other zoonotic diseases will occur. Overall, the fast-track rollout of vaccines has become the “New Normal.”

## Author contributions

VC prepared the backbone of the manuscript. VC, QY, LV, and AP wrote the original draft of the manuscript. CP, LV, VA, RB, Z-SC, QY, and VC refined the first draft. VC critically revised the manuscript for intellectually correct content. All authors contributed to the article and approved the submitted version.

## Acknowledgments

VC would like to dedicate this work to L M College of pharmacy as a part of the 75th year celebration of the college. VA would like to thank the support from the Immunology and Translational Research Group, within the Institute for Health and Sport, Victoria University Australia. VA was supported by Victoria University Research, VIC Australia. 

## Conflict of interest

The authors declare that the research was conducted in the absence of any commercial or financial relationships that could be construed as a potential conflict of interest.

## Publisher’s note

All claims expressed in this article are solely those of the authors and do not necessarily represent those of their affiliated organizations, or those of the publisher, the editors and the reviewers. Any product that may be evaluated in this article, or claim that may be made by its manufacturer, is not guaranteed or endorsed by the publisher.
